# Compensatory Structural Adaptive Modifications of Vagina in Response to Functional Demand in Goat

**DOI:** 10.1155/2014/789816

**Published:** 2014-02-23

**Authors:** Amer M. Hussin, Nazih W. Zaid, S. O. Hussain

**Affiliations:** ^1^Anatomy and Histology Department, Veterinary Medicine College, Baghdad University, Baghdad 28601, Iraq; ^2^Surgery and Obstetrics Department, Veterinary Medicine College, Baghdad University, Baghdad 28601, Iraq

## Abstract

Vaginal biopsies and smears were collected from ten adult local healthy goats. Routine histological methods were carried out on vaginal biopsies and then stained with PAS stain. The smears were stained with Methylene blue. All samples were inspected under light microscope. The present study found that many constituents of the wall of the vagina, which have an important functional role, were absent; among these were the vaginal glands, goblet cells, muscularis mucosa, and lymphatic nodules. On the other hand, vagina showed special compensatory histological mechanisms, namely, the deep epithelial folds, the well-developed germinated stratum basale, the apparent basement membrane, and the profuse defensive cells, such as neutrophils, macrophages, lymphocytes, plasma cells, and mast cells. The general stains of this study could not recognize dendritic cells although they play an important functional role. Moreover, the herein study declared also that the vaginal smears showing many adaptive cellular mechanisms among these were, the keratinization, the process of sheet formation that lines the vaginal lumen, the process of metachromasia which is related to the cellular activity in protein synthesis, keratin, and finally the presence of endogenous microorganisms. It was concluded that all the above cellular compensatory adaptive mechanisms may compensate the lacking vaginal constituents and act to raise the immune response of the vagina.

## 1. Introduction

The application of vaginal cytology as a useful tool for estrus detection in modern breeding stations has been described for some species and breeds of animals [1]. The pattern of exfoliation of vaginal cells could be used to determine the reproductive condition and by extension the ovarian functioning of the goats [2]. There were equilibrium mechanisms between living organisms and the presence of pathogens [3, 4]. Many defensive cells like neutrophils and macrophages migrate from the lamina propria to the vaginal epithelium, other cells like lymphocytes, plasma cells, and mast cells present only in the lamina propria [5].

On the other hand the cytological picture of vaginal smears was greatly affected by ovarian hormones [6]. Under the influence of estrogens, the epithelial cells synthesize and accumulate glycogen that transported in to the surface [7]. In ewes, changes in the vaginal smears were studied by [8, 9]. The high rate of fertility was associated with immune function of the reproductive tract [10]. Cells of the immune system function to prevent establishment of infection from microorganisms and to clear cancerous or damaged cells in the host [11]. Besides, keratinocytes produce immunogenic molecules and probably related to immune processes [12]. Evidence also showed that these cells are capable of producing several interleukins, colony-stimulating factors, interferons, tumor-necrosis factors, and platelets and fibroblast-stimulating growth factor [5, 13]. There was scanty information available on the exfoliative vaginal cytology of the goats [2]. However, this study was conducted to focus the light on the relation between the cellular components of the vaginal epithelium and its immune response.

## 2. Materials and Methods

Vaginal biopsies were done on ten healthy local adult goats during spring 2012. This experiment was carried out in animal's farm, College of Veterinary Medicine, Baghdad University. Samples were fixed immediately by neutral buffered formalin 10%, and routine histological processes were done. Each specimen was embedded in paraffin, and 5–7 *μ*m sections were prepared for staining with Periodic Acid-Schiff reagent (PAS) stains [14]. The vaginal smears were collected from the dorsocranial region of the vagina, using a cotton swab immersed with a saline solution. After swabs collection, they were placed on slides for future coloration using the Methylene blue method [15]. Biopsies were prepared at the same time the vaginal smears were collected, but the latter were always done first. Morphologic studies were performed using computerized camera coupled to a light microscope.

## 3. Results

**Figure 1:**
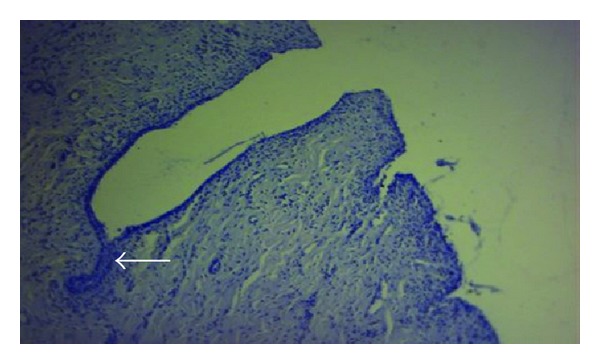
Vaginal epithelium showing deep folding; notice the incomplete gland formation (arrow). X100 PAS stain.

**Figure 2:**
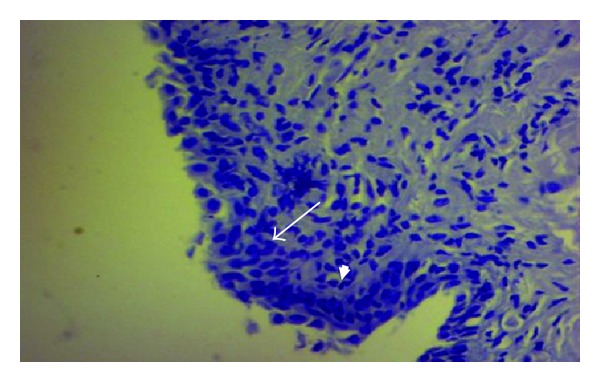
Vaginal epithelium showing the proliferated cells of stratum basale (long arrow) which covered parts of the basement membrane (arrow head). X400 PAS stain.

**Figure 3:**
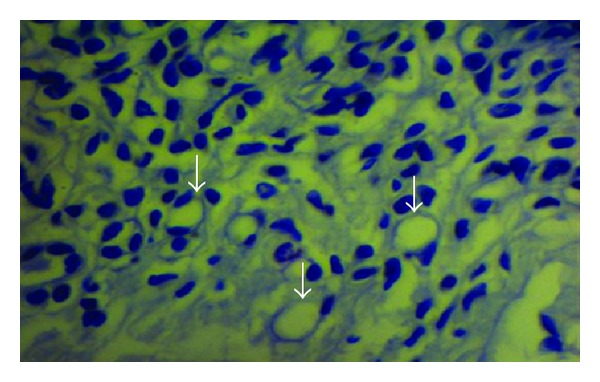
Vaginal epithelium; notice lamina propria infiltrated with different defensive cells among dilated capillaries (arrows). X400 PAS stain.

**Figure 4:**
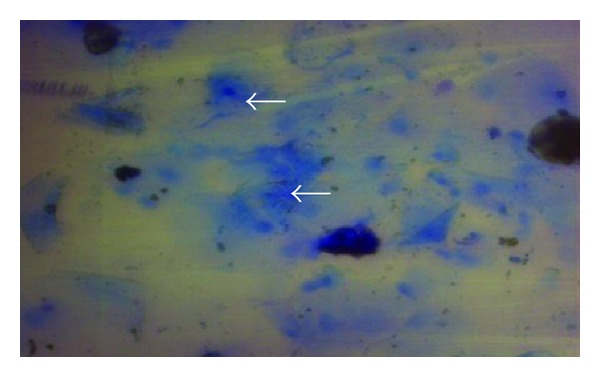
Vaginal smears; notice keratinized exfoliated apoptotic vaginal cells invaded by endogenous bacteria (arrows). X400 Methylene blue stain.

**Figure 5:**
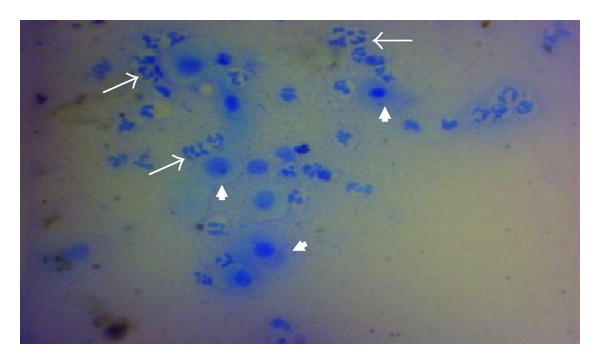
Vaginal smear; notice keratinized apoptotic cells (arrow heads) surrounded by polymorphonuclear leukocytes (long arrows). X400 Methylene blue stain.

**Figure 6:**
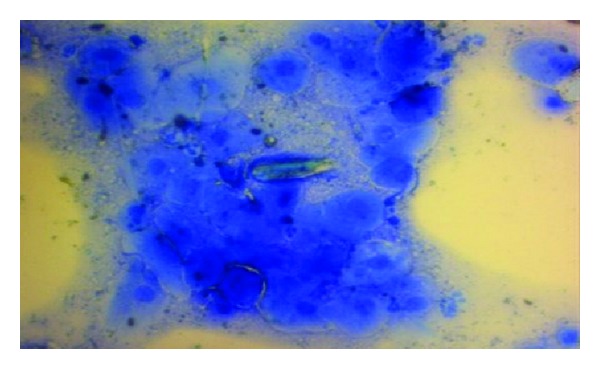
Vaginal smear, sheet formation of keratinized vaginal epithelial cells. X400 Methylene blue stain.

**Figure 7:**
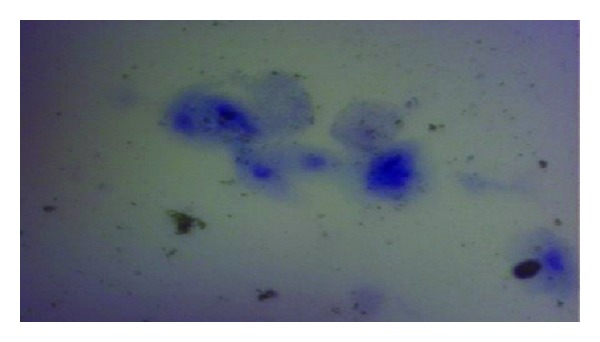
Vaginal smear, metachromasia of vaginal epithelium; notice different colours of the cells and nuclei. X400 Methylene blue stain.

This study found that, as other animals do, the vaginal wall of the goat lacks glands, muscularis mucosa, lymphatic nodules, and goblet cells. In response, many adaptive compensatory structural changes were observed, namely, cytological and cellular adaptations, leukocytes infiltration, and microflora deposition. The current results declared that the vaginal mucosa showed numerous deep folds (Figure 1), well-developed stratum basale and prominent basement membrane (Figure 2), and well-nourished lamina propria with profuse cellular elements and blood supply and presence of different immune defensive cells in the epithelium of the vagina (Figure 3). In spite of its significance, dendritic cells were not detected in this study. The findings of this study did not recognize the neutrophils in the epithelium. The keratinized apoptotic cells were attacked by endogenous bacteria (Figure 4). Polymorphonuclear leukocytes (neutrophils) move to the site of infections, encircling the affected keratinized apoptotic cells (Figure 5). This study regarded the exfoliated keratinized cells as apoptotic cells. The cytoplasm of keratinized apoptotic cells was acidophilic. Moreover, they showed the most characteristic features of apoptosis, for example, the shrinkage of cytoplasm and nuclei (pyknosis). On the other hand, the flattened polygonal vaginal cells were accumulated, coalesce to each other in order to obliterate the intercellular spaces forming broad sheets that line the vaginal mucosa (Figure 6). The herein study reported a process of metachromasia in the nuclei and cytoplasm of the exfoliated vaginal cells ranging from the basic to reddish or purple colour (Figure 7).

## 4. Discussion

The cytology of vaginal exfoliation had been described in the goat [1, 2]. The vagina serves as a passage way for oestrus flow, receives the erected penis during intercourse, and is the birth canal during parturition; this will increase the probability of occurrence of contamination in this area [16, 17].

Glands arise during fetal life by means of proliferation and invasion of the epithelial cells into the underlying tissue, followed by further differentiation [18]. The present study believed that the incomplete embryological development of the vaginal gland may lead to the formation of the deep epithelial folds which was expanded when needed and in turn participated in increasing the surface area of the vaginal epithelium. This will be raising the defensive mechanism of the epithelium that acts as a barrier separating the organism's body from its environment. The recent study was in agreement with [17] who did not refer to the presence of the mucosal goblet cells and lymphatic nodules. Samuelson [19] referred to the presence of both these constituents in the cow. The presence of a well-developed stratum basale provides the foundation for rebuilding [20]. The discontinuous appearance of the basement membrane was due to concealing part of it by the proliferating cells of stratum basale; this is in variance with [21] who reported that the basement membrane often forms continuous sheets but may also be discontinuous at some locations. Basement membrane provides physical support for tissue, influences cell proliferation, migration, and differentiation, and is thus implicated in biological processes such as development, tissue maintenance, regeneration, and repair; in addition, the basement membrane acts also as reservoir of growth factors, enzymes, and plasma proteins [18]. Moreover, from the functional point of view in other systems, the muscularis mucosa of digestive system produces local movements of the mucosa leading to enhancing the contact between epithelium and the contents of the lumen, for example, twitching of this muscle layer dislodges food particle, that have been adhered to the mucosa. Muscularis mucosa aids also in transportation of glands secretions [22]. In this study, the function differs and there is no need for the presence of such mechanism; instead, there are great amounts of elastic fibers to increase expansion of the vagina leading to enhancing the parturition [23].

Much controversy had been reported about the basement membrane in the vagina. Many authors neglected the referring to the basement membrane [19]. Other authors did not refer clearly to the presence or absence of such membrane [24] who stated that some epithelia rest on a basement membrane, while [18] reported that all epithelial tissues rest on a basement membrane. Bajpai [25] confirms that the epithelium rests directly on a lamina propria. Our hypothesis declared that where there is no heavy cellular infiltration, the basement membrane was apparent, whereas in other areas where the basement membrane was hidden by heavy cellular infiltration, this membrane was obscured. This phenomenon was misleading many authors. Copenhaver et al. [26] was the only one who referred to the presence of such membrane in the human's vagina. This result confirms the presence of such basement membrane in the vagina of the goat.

The abundant engorged blood vessels present in the underlying tissue demonstrating the rich blood supply to the vagina. These blood vessels supply metabolites, vitamins, growth factors, antibodies, and phagocytized cells [17]. Heavy infiltration of neutrophils in the vaginal lumen was observed as a first line of defense. This coincided with [8, 9, 27] who stated that the presence of neutrophils in ewes increases the opportunity of phagocytizing any bacteria and small particles. Increase in neutrophils infiltration during diestrus is usually consistent with evidence of mating activity or arrival of diestrus [28]. The present study was in variance with [29] who demonstrated that the leukocytes were present among the epithelial cells. Macrophages migrate in response to chemotactic stimuli; they phagocytize and kill bacteria [30]. Reference [17] reported that lymphocytes are found only in the stroma and not in the epithelium of ewes; this is similar to the findings of the present study. Besides, plasma cells secrete large quantity of antibodies into the general circulation. Mast cells function in inflammatory response, innate immunity, and tissue repair [31]. These cells were found around the blood vessels. In spite of its importance in immune defense, this study was incapable of recognizing the dendritic cells because they are hard to detect in routine staining [24].

The importance of keratinization was to protect the vaginal epithelium from invasion of microorganism. This finding coincided with [32] who reported that keratinization activates immunization. Samuelson [19] and Mescher [24] stated that cells undergoing apoptosis tend to be isolated from one another in general population with shrinkage of cellular and nuclear volume (pyknosis). This is similar to the finding of the present study as the apoptotic cells become isolated from their population and then shed into the lumen of the vagina. Acidophilia of the cytoplasm of keratinized apoptotic cells may be due to the increase of the number of mitochondria to aid in lifting their junctional attachments with the neighboring cells. This partly confirms [33].

The herein result demonstrated that the cellular sheet formation and the intercellular communications have an important role in raising the cellular adaptive response, by obliterating the spaces between the vaginal epithelial cells to prevent the penetration of materials and invasion of pathogens between the cells and the underlying tissue. This agrees with the finding of [34] about keratinization in the vagina of ewe.

Endogenous bacteria metabolize the glycogen into lactic acid leading to decreasing the pH of the vagina and protecting it from pathogenic bacteria. This confirms the finding of [32, 34, 35].

This result found also that the cytoplasm and nuclei of the vaginal epithelium undergo metachromasia, through which the tissue colour shifts from blue to red or purple. High concentrations of rough endoplasmic reticulum exhibit cellular metachromasia which is important in synthesis of the protein and keratin during the process of keratinization. This statement is in accordance with the result of [36] which stated that metachromasia was influenced by the ovarian activity.

The recent study referred to the relationship between the affected keratinized apoptotic vaginal epithelial cells and the phagocytizing neutrophils. This is confirmed by [37] which reported a positive relation between the cornified and leukocyte cells in the vagina of the rat and proposed that the influx of the leukocytes could be a response to the increased bacterial flora that associated with the cornified desquamated cells of the oestrus phase.

## 5. Conclusion

Although the relation between structure and function is now well understood, much remains to be discovered about how the cellular components of any organ interact with one another. As structure follows function, the special location and the type of function of the vagina lead to deterioration and adaptation of its components to overcome the functional need. Cells and tissues form and cope according to their location and functional demand as the same cell type can exhibit different characteristics and behaviors in different regions and circumstances.
